# Disability and living arrangements among older immigrants in the US: evidence from the American Community Survey

**DOI:** 10.1093/haschl/qxag043

**Published:** 2026-03-04

**Authors:** Mahir Rahman, Momotazur Rahman

**Affiliations:** Brown University, Providence, RI 02912; Brown University, Providence, RI 02912

**Keywords:** aging, health services, long-term services and supports, immigrant health, living difficulty

## Abstract

**Introduction:**

The demographic composition of the US older adult population as individuals who are immigrants continues to rise.

**Methods:**

Using data from the American Community Survey, this study examines trends, health status, and living arrangements among U.S.-born and immigrant older adults.

**Results:**

The share of immigrants who are older adults increased from 10% in 2001 to over 15% in 2023, with Latin America and the Caribbean (LAC), Asia, and Africa driving much of this growth. We found significant differences in health and living arrangements across regions of birth. Immigrants from LAC reported fewer health difficulties. Immigrants from other origins have comparable health difficulties as the native-born. Immigrants from Africa and Asia were less likely to reside in nursing homes but more likely to live with offspring. Native-born older adults with independent living difficulties were disproportionately more likely to reside in group quarters.

**Conclusion:**

These findings underscore the importance of accounting for heterogeneity in the ageing immigrant population and addressing their growing presence's cultural, economic, and policy implications.

## Introduction

With the ageing of the baby boomer generation and rising life expectancy, the United States is undergoing a rapid demographic shift toward an older population. Older adults made up about 13% of the U.S. population in 2010, increasing to roughly 17% by the early 2020s, and are projected to reach around one-fifth of the population by 2050. This surge results in a growing number of individuals with cognitive and functional impairments, which is driving unprecedented demand for long-term services and supports (LTSS). Historically, formal LTSS in the U.S. were delivered mainly in institutional settings such as nursing homes.^1^ However, soaring nursing home costs and the clear preference of most older adults to age in their own homes or communities have prompted a pivot toward expanding home- and community-based LTSS.^2,3^ As we confront this monumental challenge, there is a risk that policy discussions focus narrowly on medical needs or cost containment while overlooking the heterogeneity among older adults—differences in social, cultural, and family circumstances that can shape an individual's long-term care preferences and choices.^4^

One critical factor influencing LTSS demand is the evolving demographic composition of the older adult population, particularly the growing share of immigrants. The United States is home to the largest immigrant population in the world, with 47.8 million immigrants as of 2023.^5^ This growing diversity introduces cultural and social differences that can shape long-term care preferences, attitudes, and behaviors. Yet, despite this demographic shift, little is known about the health differences and LTSS use between U.S.-born and immigrant older adults. Understanding these differences is essential for designing equitable and effective policies to meet the needs of this increasingly diverse population. Policymakers today face critical decisions about how much to further “rebalance” the LTSS system toward community-based care vs sustaining institutional capacity, and how to support the unpaid family and friend caregivers who continue to provide the majority of elder care.^6,7^

Immigrants differ from U.S.-born individuals in several ways that may influence their health. Factors such as childhood investments in health, eligibility restrictions for public programs,^8^ employment in lower-income and high-risk jobs,^9^ and experiences of healthcare discrimination^10^ contribute to disparities in health outcomes and access to care. Immigrant populations differ in cultural attitudes such as familial duty,^11,12^ language preferences,^13^ and culturally congruent services^14^ that shape immigrant experiences and preferences for long-term care. Additionally, immigrant older adults may differ in economic capacity to afford care, and enrollment in Medicaid or long-term care insurance that pays for formal LTSS. As healthcare utilization increases with age, it is crucial to examine the unique needs and access challenges of older immigrant adults.

This study addresses these gaps by using data from the American Community Survey (ACS)^15^ to examine differences in health and living arrangements between U.S.-born and immigrant older adults. Specifically, we make three key contributions. First, we document the temporal and geographic variation in the share of the older adult population who are immigrant. Second, we assess differences in health status, focusing on the presence of cognitive and functional difficulties. Third, we explore how these difficulties influence living arrangements, including cohabitation with offspring and nursing home residency. By addressing these questions, this study aims to provide insights into the evolving needs of the ageing U.S. population and inform strategies for equitable long-term care provision.

## Study data and methods

### Data

This study utilized data from the ACS,^15^ an ongoing national survey conducted by the U.S. Census Bureau. The ACS collects detailed demographic, social, economic, and housing information from a representative sample of more than 3.5 million households annually. It is a key resource for policymakers, researchers, and businesses, providing essential insights to inform decisions at local, state, and national levels. The survey covers a wide range of topics, including age, education, income, employment, migration, household structure, and health insurance coverage. Notably, the ACS includes individuals residing in group quarters, such as nursing homes, correctional facilities, and college dormitories, and records respondents’ place of birth. For this study, ACS data was accessed through the IPUMS USA database.

### Study cohort

The study population consisted of individuals aged 65 and older. For trend analyses, we utilized ACS data spanning from 2001 to 2023. The remaining analyses focused on data from 2023, which included 788 193 individuals residing in 572 523 households or group quarters. All analyses applied the person weights provided by the ACS to ensure that the statistics accurately represent the entire U.S. older adult population, totaling 59 325 594 individuals.

### Main explanatory variable

The primary explanatory variable was the region of birthplace. For the geographic variation analysis, this variable was treated as binary, indicating whether the individual was born in the United States or abroad. To explore heterogeneities among immigrants from different parts of the world, we categorized all individuals into five groups based on their country of birth (as defined by ACS/IPUMS birthplace codes): USA, Africa, Asia, Latin America and the Caribbean (LAC), and Europe/Canada/Australia. The category of LAC includes individuals born in Mexico, Central America, the Caribbean/West Indies, and South America. For these analyses, the variable was used as a categorical variable, with the USA as the reference category.

### Outcomes

To assess differences in health status between immigrant and U.S.-born older adults, we examined three ACS disability indicators: cognitive, ambulatory (mobility), and independent living difficulty, each coded as a binary measure. Cognitive difficulty indicates serious difficulty concentrating, remembering, or making decisions because of physical, mental, or emotional condition. Ambulatory difficulty indicates serious difficulty walking or climbing stairs. Independent living difficulty indicates a physical, mental, or emotional condition lasting at least 6 months that makes it difficult or impossible to perform basic activities outside the home alone, such as shopping or visiting a doctor's office, and excludes temporary conditions (eg, broken bones).

To analyze differences in living arrangements, we used a three-category outcome: residing in a household without offspring, residing in a household with offspring, and residing in a group quarter.

### Control variables

Our control variables included age, sex, marital status, race, ethnicity, enrollment in Medicare, Enrollment in Medicaid, education and veteran status. We controlled for residential state/county fixed effects to account for difference in long-term care policies and unobserved heterogeneity by geographic area. Additional details on how these variables were coded are provided in the Appendix Table S1.

### Analysis

We began by examining trends in the share of older adults in the U.S. who were immigrant from Africa, Asia, LAC, and Europe/Canada/Australia from 2001 to 2023. To visualize these trends, we stacked bars that represent the share of the older adult population in the US with different places of birth, such that the entire bar for a specific year represents the total share of the immigrant population.

To illustrate the geographic variation in immigrant older adults, we calculated the share of immigrant individuals within the older adult population by the residential state for 2023 and mapped these values. Additionally, as a supplemental analysis, we identified states with the largest growth in immigrant populations by plotting the share of immigrant older adults in 2023 against their respective shares in 2001.

To assess differences in health status across regions of birth, we performed regression analyses examining the association between having a specific difficulty (cognitive, ambulatory, or independent living) and region of birth (using the U.S. as the reference category). The models controlled for county-fixed effects, age, sex, marital status, race, health insurance status, education, and veteran status. We plotted the excess risk-adjusted share of the population with specific health difficulties by foreign region of birth relative to US-born. To assess whether the excess outcomes among immigrants vary by age we also estimate these models by interacting region of birth categories with age and age squared.

Finally, we analyzed differences in living arrangements between immigrant and U.S.-born older adults, focusing on how these differences vary by the presence of any independent living difficulty. For this, we estimated a multinomial logit model of living arrangement outcome: residing in a household without offspring vs residing in a household with offspring vs residing in a group quarter. The regressors include interactions between the presence of independent living difficulty and region of birth, along with the control variables and state fixed effects. We then calculated margins to estimate the difference risk-adjusted probabilities of a specific living arrangement between individuals with and without independent living difficulties for each region of birth.

All statistical analyses were conducted in Stata and were weighted using frequency and analytic weights to ensure national representativeness.

### Limitations

This study has several limitations that should be acknowledged. *First*, this study utilized survey data from the ACS, which may carry inherent biases associated with survey data collection and self-reporting despite the application of analytic weights. *Second*, our region-of-birth categories are necessarily broad and should be interpreted as descriptive proxies rather than conceptually homogeneous immigrant groups. For instance, Europe, Canada, and Australia were grouped primarily based on their high-income status, though countries within these regions—and similarly within Asia—exhibit considerable heterogeneity in terms of per capita income. Importantly, each region aggregates countries with very different migration pathways, socioeconomic profiles, and caregiving norms, and therefore may mask meaningful within-region heterogeneity in health and living arrangements. In particular, the “LAC” category combines Mexico, Central America, the Caribbean, and South America, groups that likely differ in immigrant selection, return migration, and access to diagnosis and long-term care services. Future work should disaggregate these regions when sample sizes and study aim permit. *Third*, ACS items may be reported by a single household respondent on behalf of other household members, which can introduce measurement error if the respondent has incomplete knowledge of another person's functional or cognitive limitations. Importantly, such reporting may capture differences in awareness, diagnosis, language, stigma, or cultural norms around reporting difficulties, which could vary by nativity and region of birth. *Finally,* this study aimed to describe differences in health outcomes among individuals from various origins rather than establish causal relationships. While we controlled for demographic variables and region fixed effects, measurement error and unobserved confounding correlated with immigration status may still bias our estimates.

## Results

**Figure 1.**
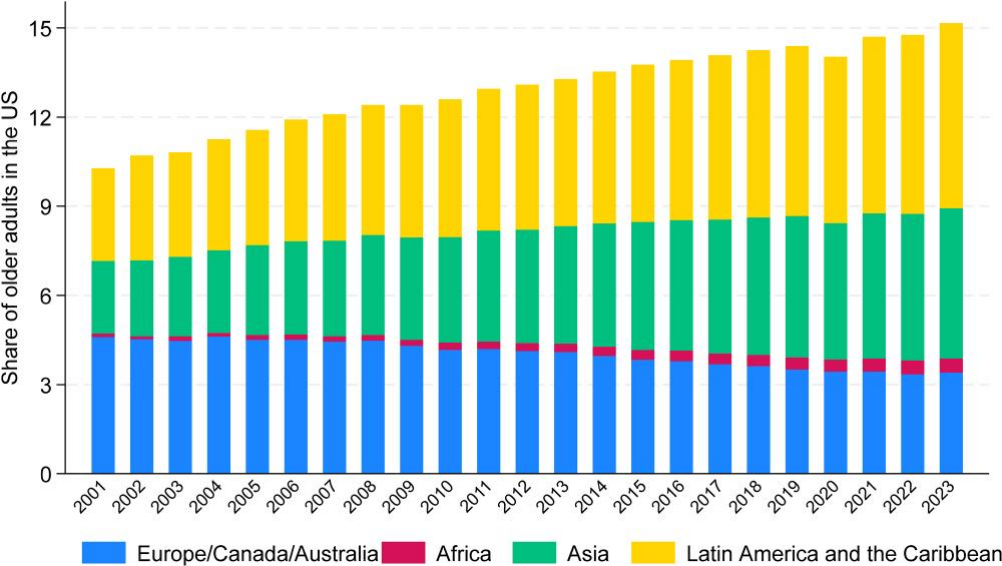
Trends in the share of age 65+ US population by place of birth. Source: Authors’ calculation based on the ACS using the age 65+ population.

Figure 1 illustrates the growing diversity of the U.S. older adult population (aged 65 and older) by documenting the share of older adults who are immigrant from four global regions between 2001 and 2023. Over this period, the proportion of immigrants in the older U.S. population steadily increased from 10% in 2001 to over 15% in 2023. This growth reflects significant shifts in the countries of origin among older immigrants. The most notable increase is among those from LAC, who now make up approximately 6% of the older population, followed by Asia, contributing around 5%. African-born immigrants, although comprising a smaller share, have seen their representation rise nearly fourfold, from 0.12% in 2001 to 0.47% in 2023. In contrast, immigrants from Europe, Canada, and Australia—who made up the largest group of older immigrant individuals in 2001 at 4.6%—have decreased to 3.4% by 2023.

**Figure 2.**
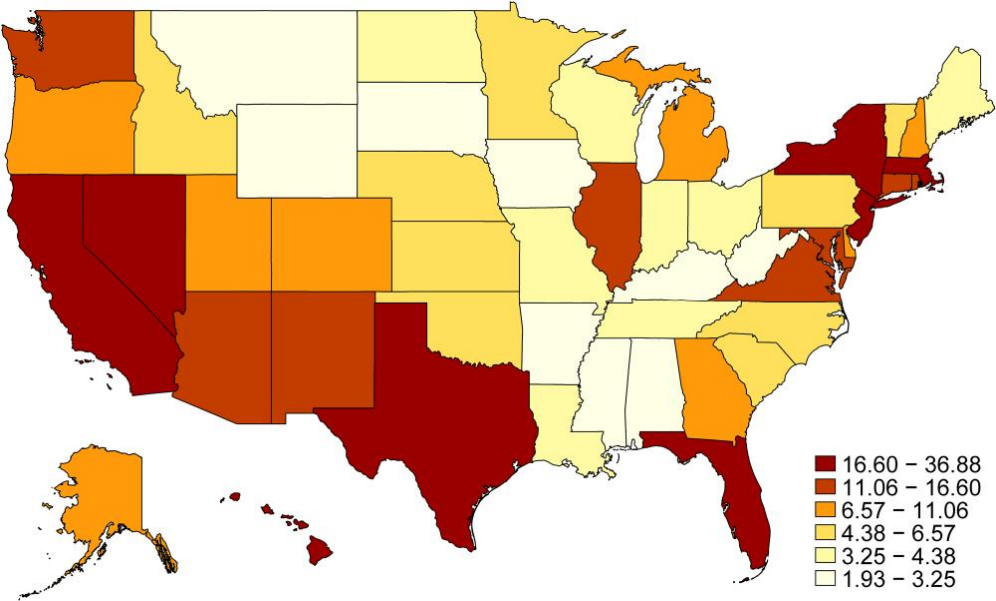
Distribution of older adult immigrants by state in 2023. Source: Authors’ calculation based on the ACS using the age 65+ population in 2023.

Figure 2 highlights the geographic variation in the share of older adults (aged 65 and older) who are immigrants across U.S. states in 2023. California has the highest proportion of older adult immigrants, at 36.9%. Several other states, including New York (28.6%), Hawaii (24.0%), Florida (23.6%), and Nevada (22.4%), also report immigrants exceeding 20% of their older adult population. Texas (18.8%) and Massachusetts (17.2%) follow closely behind. In contrast, the states with the smallest immigrant older adult populations are primarily concentrated in the Midwest and Southern regions, with Mississippi (1.9%), Alabama (3.2%), and West Virginia (2.1%) reporting the lowest shares. This data underscores the considerable variation in the distribution of older adult immigrants across the country, with coastal and Sun Belt states generally hosting higher proportions of immigrant older adults. It is additionally critical to examine the increase in share of immigrants by state over the past 20 years. Since 2001, California and New York experienced the largest increase by percentage of immigrants to total state population (see Appendix Figure S1). We additionally note that since 2001 the share of older African immigrants with residence in the US for 5 years or more has substantially decreased, that of European, Australian, and Canadian older immigrants has stayed relatively constant, and the share of African and Asian older immigrants living in the US for five plus years has slightly decreased (see Appendix Figure S2).

Appendix Table S1 summarizes the differences in socio-demographic characteristics, health status, and living arrangements between US-born and immigrant older adults. African immigrants are the youngest group, with 73.1% aged 65-75, while European, Canadian, and Australian immigrants are the oldest, with only 55.0% in that age range. Gender distributions also vary, with Africa being the only region where most older adult immigrants are male with a 47.8% female population. In contrast, immigrants from other regions, including Asia and LAC, have a majority female population (57.5%).

Race, ethnicity, and education levels also show notable contrasts. African immigrants are the most racially diverse, with 31% identifying as White and 57% as Black. Conversely, 96% of immigrants from Europe, Canada, and Australia are White. Immigrants from LAC have the lowest levels of education, with 41.6% lacking a high school diploma. In contrast, African and Asian immigrants are the most highly educated, with 46.8% and 39.4% holding a college degree, respectively. Native-born Americans sit between these extremes, with 31.5% having completed college. Health insurance and veteran status differ sharply between groups. African immigrants have the lowest Medicare coverage (83.6%) while native-born older adults have the highest Medicare coverage (96%) and veteran status (14.7%). Additionally, immigrants from LAC have the highest Medicaid enrollment (32.0%) and the lowest amount who served as a veteran (3.0%).

In unadjusted comparisons, immigrants from Africa exhibit the lowest prevalence of disability, while those from LAC have the highest. Specifically, 7.5% of African immigrants report cognitive difficulty, 18.5% report ambulatory difficulty, and 13.8% report independent living difficulty. In contrast, these rates are 11.1%, 23.7%, and 17.1%, respectively, for immigrants from LAC. However, these figures do not account for differences in demographic and socioeconomic factors, such as age, education, or veteran status, which may influence disability rates.

**Figure 3.**
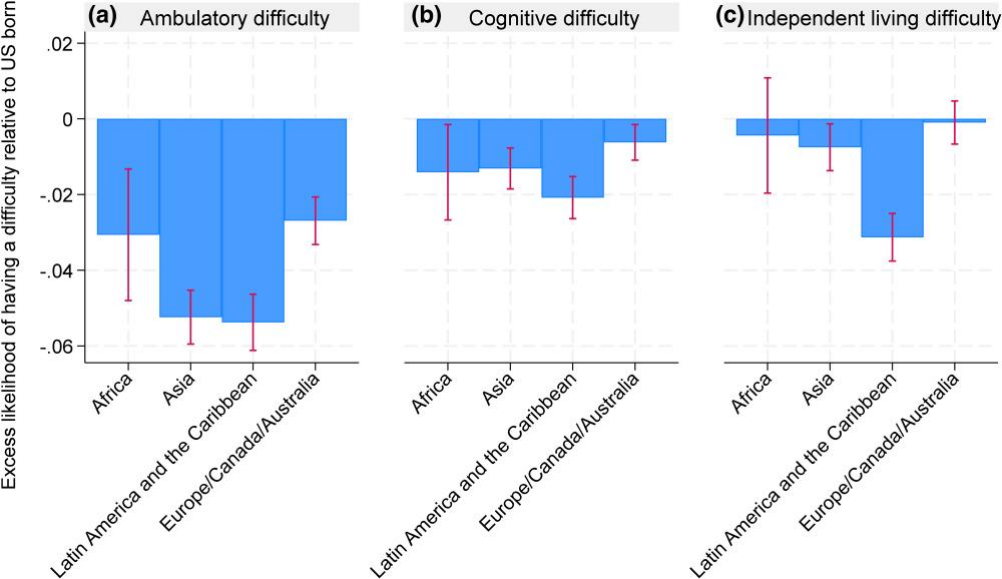
Difference in the prevalence of specific difficulties among older adults with different places of birth relative to the US born in 2023. Source: Authors’ calculation based on the ACS using the age 65+ population in 2023. Note: Estimates are based on linear regression of having a specific difficulty onto control variables and county fixed effects. Three panels are based on three separate regressions. Detailed regression results are presented in Appendix Table S2.

Figure 3 presents adjusted differences in disability by region of birth relative to U.S.-born older adults, accounting for age, education, veteran status, and insurance coverage. The regression model used for risk adjustment is detailed in Appendix Table S2. After adjustment, all immigrant groups were less likely than U.S.-born older adults to report ambulatory and cognitive difficulties. Immigrants from LAC had the lowest adjusted rates of difficulty: they were 5.4% points (95% CI: −6.1 to −4.6) less likely to report ambulatory difficulty, 2.1% points (95% CI: −2.6 to −1.5) less likely to report cognitive difficulty, and 3.1% points (95% CI: −3.6 to −2.5) less likely to report independent living difficulty. While U.S.-born older adults generally had higher rates of cognitive and ambulatory difficulties, differences in independent living difficulty were only significant when compared with Immigrants from LAC. In models with age and region of birth interaction, immigrants exhibited lower prevalence of cognitive, ambulatory, and independent living difficulty at age 65 and health advantages declined steadily with age (Appendix Figure S3). Immigrants have lower ambulatory difficulty at all age but had higher cognitive and independent living difficulties at age 85 and older.

Appendix Table S1 highlights notable differences in living arrangements among older adults by region of birth. Immigrants from Africa (40.3%), Asia (38.7%), and LAC (41.5%) are significantly more likely to reside in households with their offspring compared with U.S.-born older adults (14.5%) and those from Europe, Canada, and Australia. In contrast, native-born older adults are slightly more likely to live in group quarters (3.2%) than immigrants from all other regions, where the rates range between 1% and 2%. While the overall prevalence of group quarters residence is low across all groups, this pattern suggests a greater reliance on family-based living arrangements among many immigrant communities.

**Figure 4.**
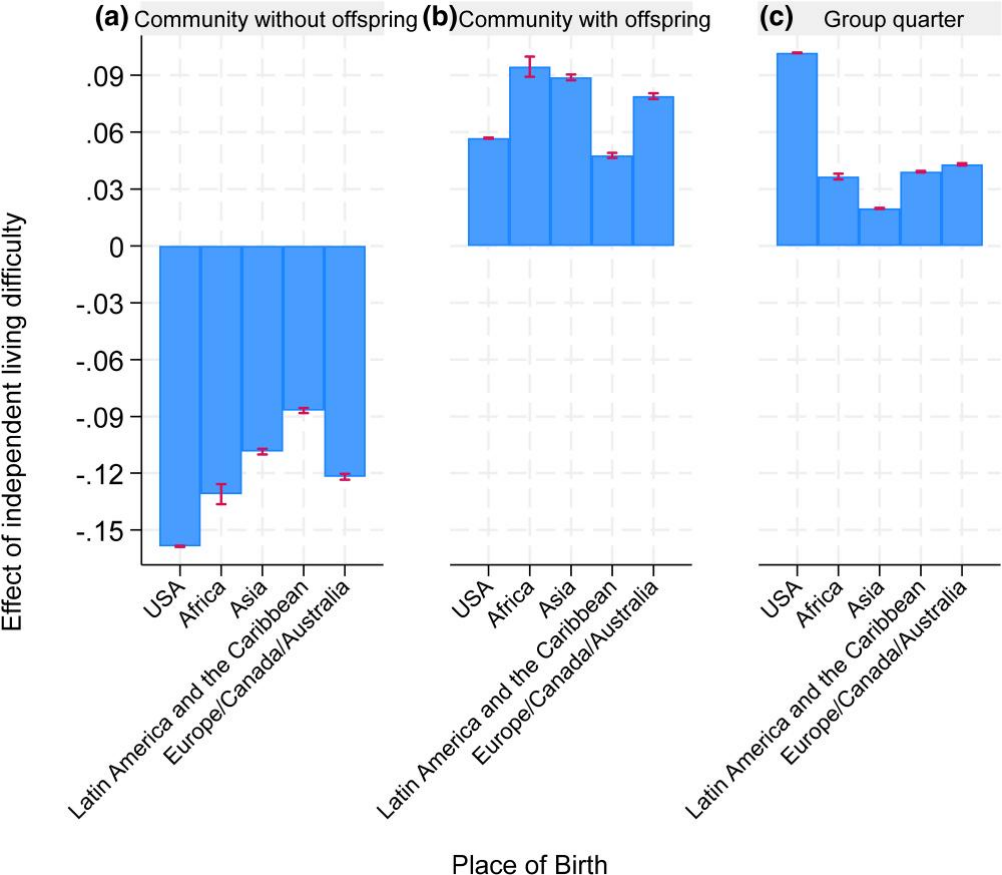
Effect of independent living difficulties on the living arrangement for older adults in the US in 2023 by place of birth. Source: Authors’ calculation based on the ACS using the age 65+ population in 2023. Note: Estimates are based on a multinomial logit regression of regression of living arrangement onto control variables and state fixed effects. Three panels are based on margins following regression estimation for three categories of the outcome. Detailed regression results are presented in Appendix Table S3.

Figure 4 presents risk-adjusted differences in living arrangements by region of birth and independent living difficulty (see Appendix Table S3 for model details). Across all groups, having an independent living difficulty is associated with a higher likelihood of living with offspring or in a group quarter, and a lower likelihood of living independently without offspring. However, the magnitude of these changes varies by nativity. Among U.S.-born older adults, the presence of an independent living difficulty reduces the probability of living independently without offspring by 15.9% points, while increasing the likelihood of co-residing with offspring by 5.7% points (95% CI: 5.6-5.8) and residing in group quarters by 10.2% points (95% CI: 10.1-10.2). For African and Asian immigrants, the presence of an independent living difficulty increases the likelihood of living with offspring by 9.4% points (95% CI: 8.9-10.0) and 8.9% points (95% CI: 8.7-9.0), respectively. In contrast, their likelihood of residing in group quarters increases by only 2% to 4% points—substantially less than that of native-born older adults—highlighting differing cultural norms and caregiving practices.

## Discussion

Our analysis reveals significant demographic, geographic, and socioeconomic differences among the U.S. older adult population based on place of origin. Over the past two decades, the proportion of immigrant older adults has steadily increased, particularly among immigrants from LAC, Asia, and Africa, while the share of those from Europe, Canada, and Australia has declined. Coastal and Sun Belt states, such as California, New York, and Florida, host the highest concentrations of older adult immigrants. Differences in health status and living arrangements are also notable: immigrants from LAC report fewer health difficulties, and immigrants from Africa and Asia are more likely to live with their offspring compared with native-born older adults. Meanwhile, native-born older adults with independent living difficulties are more likely to reside in group quarters.

Our findings highlight the heterogeneity among older adult immigrants in the U.S., with differences in demographic, socioeconomic, and health characteristics depending on region of origin. Immigrants from Africa, Asia, and LAC are generally younger and more likely to reside with offspring, while those from Europe, Canada, and Australia tend to be older. Educational attainment also varies, with African and Asian immigrants having the highest rates of college graduation and immigrants from LAC the lowest. These findings underscore the importance of disaggregating immigrant populations by place of origin, as their experiences and outcomes are far from monolithic. This aligns with prior studies that emphasize variation in socioeconomic status, access to healthcare, and social support across immigrant groups, while our study provides additional insights into how these disparities persist into old age and influence health and living arrangements.

Our findings of lower rates of ambulatory and cognitive difficulties among immigrants are consistent with documented immigrant health differences that reflect selection operating at multiple stages. Immigrants are often positively selected on health and other traits that facilitate migration,^16^ and additional selection may occur over time through selective survival and differential return migration (ie, individuals with worsening health may be more likely to return to their country of origin),^17^ which can mechanically increase the apparent health of immigrants who remain in the United States at older ages. However, the health advantage is less pronounced for independent living difficulties, which may reflect better technological or social support for native-born older adults. Importantly, immigrants’ advantages in cognitive, ambulatory, and independent living difficulty are largest at age 65 and steadily decline with advancing age (Appendix Figure S3), a pattern that is consistent with the idea that initial selection advantages may erode as health shocks accumulate and as exposures and constraints in the U.S. increasingly shape late-life disability trajectories.^16,18^

Our results show that immigrants from LAC (most of whom are Hispanic) have the lowest levels of health difficulties among older adults. However, we cannot infer whether these results are due to a broader immigrant health advantage that can arise from a combination of entry selection, selective retention/return migration, and cohort composition or the Hispanic paradox.^19,20^ Moreover, the same descriptive patterns could also be influenced by measurement and access, because these ACS items reflect self/proxy-reported difficulties that may be shaped by differential diagnosis, language barriers, or stigma. Taken together, these considerations suggest that cross-sectional differences at older ages should be interpreted as descriptive evidence consistent with selection and convergence mechanisms, not as evidence of stable protective factors.

Our findings have direct implications for how states and the federal government plan for LTSS as the older immigrant population grows. Lower nursing home residence and higher co-residence with offspring among several immigrant groups may signal a shift in the location of care from institutions to households. This may explain in declining occupancy rates and nursing home closures partially.^21^ While earlier studies reported increased nursing home use among Hispanic and Asian populations,^22^ our findings suggest that this growth is due to the increase in size of this population but not their higher use of institutional care.

Nevertheless, the lower use of institutional long-term care should not be interpreted as lower need for LTSS; instead, it may reflect a mix of preferences and constraints (eg, affordability, eligibility, and availability of culturally and linguistically congruent services). To the extent that family care substitutes for publicly financed institutional care, the financing burden is partially shifted from Medicaid and providers to unpaid caregivers, with potential downstream consequences for caregiver health, labor supply, and intergenerational stress. These patterns argue for policy emphasis on strengthening home- and community-based services (HCBS) and caregiver supports—such as respite care, caregiver training, navigation assistance, and culturally appropriate home care.

The results also underscore how immigrant ageing intersects with Medicare and Medicaid eligibility and access to care. Older immigrants have heterogeneous work histories, legal statuses, and insurance pathways that shape their ability to obtain timely diagnosis and treatment and to qualify for Medicare-covered post-acute services and Medicaid-financed LTSS—factors that may influence both reported difficulties and observed patterns of institutional vs community care. At the same time, the findings that immigrants’ health advantages are largest at age 65 and decline with age imply that service needs may rise disproportionately as immigrant cohorts reach towards the end of life, reinforcing the importance of early outreach, language access, and culturally competent screening and care management to prevent avoidable functional decline. Taken together, these findings point to a need for LTSS policy that explicitly accounts for the growth and heterogeneity of older immigrants—supporting family-based care when it is desired.

## Conclusion

Our study provides further transformative trends in immigrant populations in the United States. Few studies have highlighted recent developing trends among all major immigrant groups and commented on this key heterogeneity. Our findings also supported literature surrounding key concepts including the healthy immigrant effect, Hispanic paradox, nature of family caregiving among immigrants, and changing demand for nursing home care.

In the future, it will be imperative to delve further into the heterogeneity of the increasingly ageing immigrant population. As political and economic contexts change both within and outside of the United States following a new presidential administration, it will be even more important to understand the heterogeneity of the immigrant populations and understanding ageing concerns.

## Supplementary Material

qxag043_Supplementary_Data
