# Supplementary material for: Disability and living arrangements among older immigrants in the US: evidence from the American Community Survey
Source: Health Aff Sch. 2026 Mar 4;4(3):qxag043. doi: 10.1093/haschl/qxag043 (PMC13008007; doi:10.1093/haschl/qxag043)
Supplement: qxag043_Supplementary_Data [file qxag043_supplementary_data.zip › Statistical Appendix.pdf]

Statistical Appendix

Appendix Figure 1: Share of immigrant among older adults in 2001 and 2023 by state

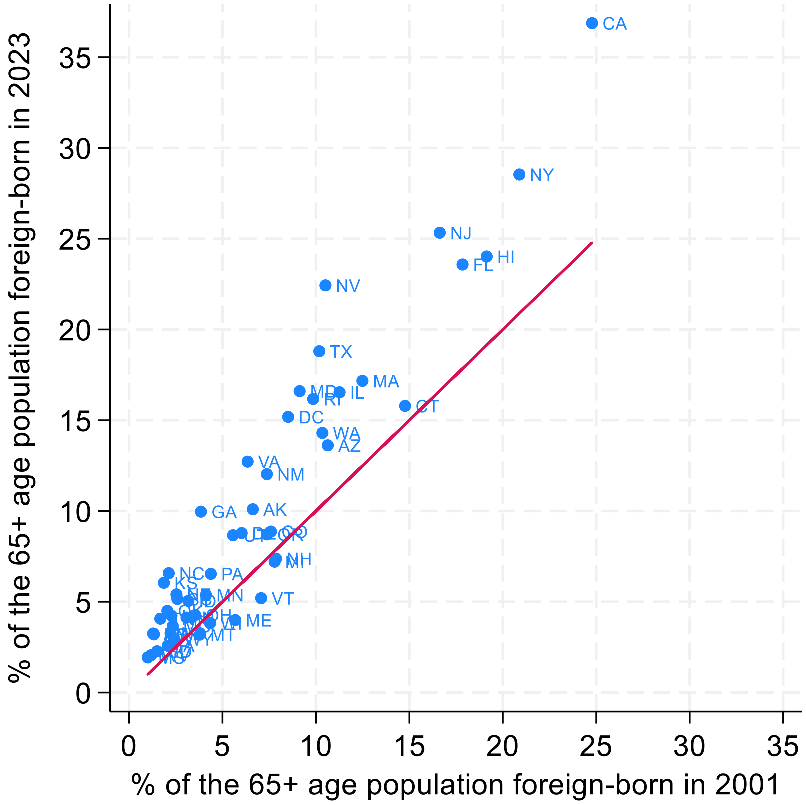

Appendix Figure 2: Share of individuals residing in the US for 5+ years by place of birth

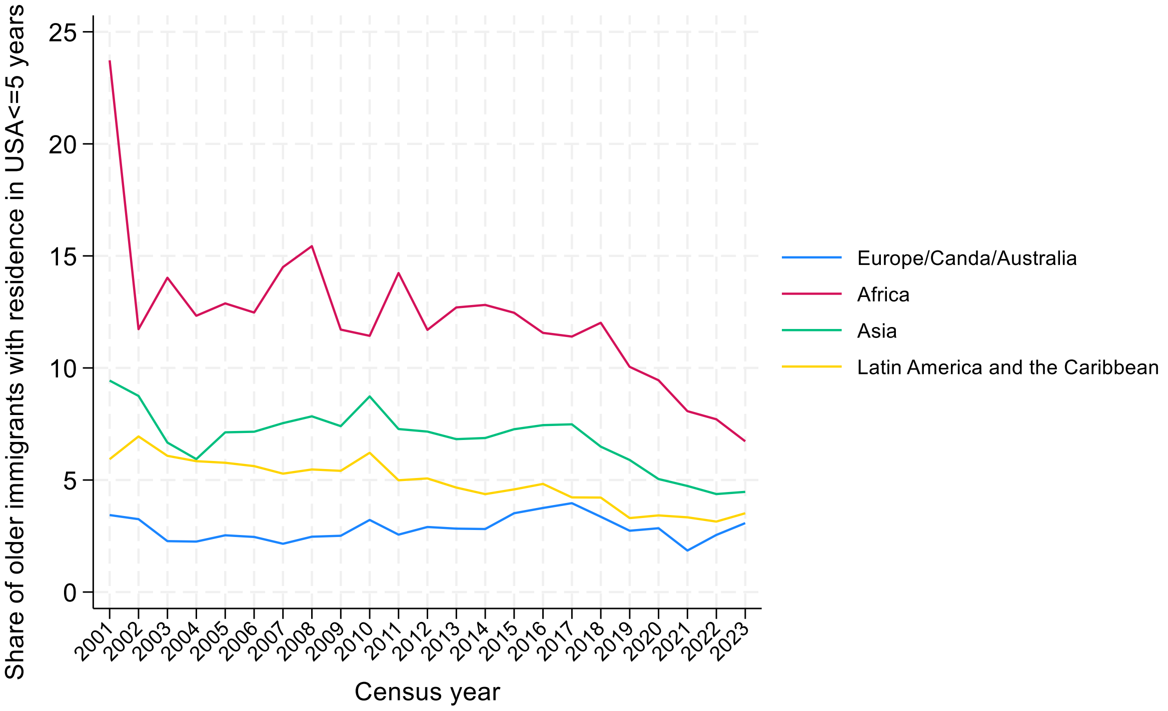

Appendix Table 1: Characteristics of older adults in the US in 2023 by place of birth

	Africa	Asia	Latin America and the Caribbean	Europe, Canada, Australia	USA
N	2,844	33,564	36,083	24,908	690,794
Weighted N	280,598	2,998,624	3,693,412	2,021,104	50,331,856
Age 65-75	73.1%	65.0%	66.8%	55.0%	63.4%
Age 76-85	21.4%	27.0%	25.5%	32.4%	28.0%
Age 85 and above	5.6%	8.1%	7.7%	12.6%	8.6%
Female	47.8%	55.9%	55.8%	57.5%	54.6%
Married	57.9%	65.3%	53.9%	57.8%	55.8%
Race: White	30.0%	11.9%	11.4%	96.4%	82.7%
Race: black	59.5%	0.1%	13.0%	0.3%	9.8%
Race: Other	10.5%	88.0%	75.5%	3.3%	7.5%
Ethnicity: Hispanic	0.7%	0.4%	81.8%	1.9%	5.0%
Education: Less than high school	11.8%	19.6%	41.6%	11.2%	6.9%
Education: High school	24.4%	26.8%	30.7%	34.3%	40.3%
Education: Some college	17.0%	14.2%	12.4%	18.8%	21.4%
Education: College Graduate	46.8%	39.4%	15.3%	35.7%	31.5%
Veteran	3.3%	3.2%	3.0%	6.6%	14.7%
Enrolled in Medicaid	21.8%	27.7%	32.0%	16.1%	13.5%
Enrolled in Medicare	83.6%	90.8%	88.2%	92.5%	96.0%

Any cognitive difficulty	7.5%	9.7%	11.1%	9.0%	8.8%
Any ambulatory difficulty	18.5%	19.7%	23.7%	20.3%	22.0%
Independent living difficulty	13.8%	17.1%	17.1%	16.4%	14.3%
Residing in a household with offspring	40.3%	38.7%	41.5%	17.2%	14.5%
Residing in a group quarter	1.4%	1.1%	1.6%	2.1%	3.2%

Source: Authors' calculation based on the American Community Survey using the age 65+ population in 2023.

Note:

Appendix Figure 3: Difference in the prevalence of specific difficulties among older adults with different places of birth relative to the US born in 2023, by age

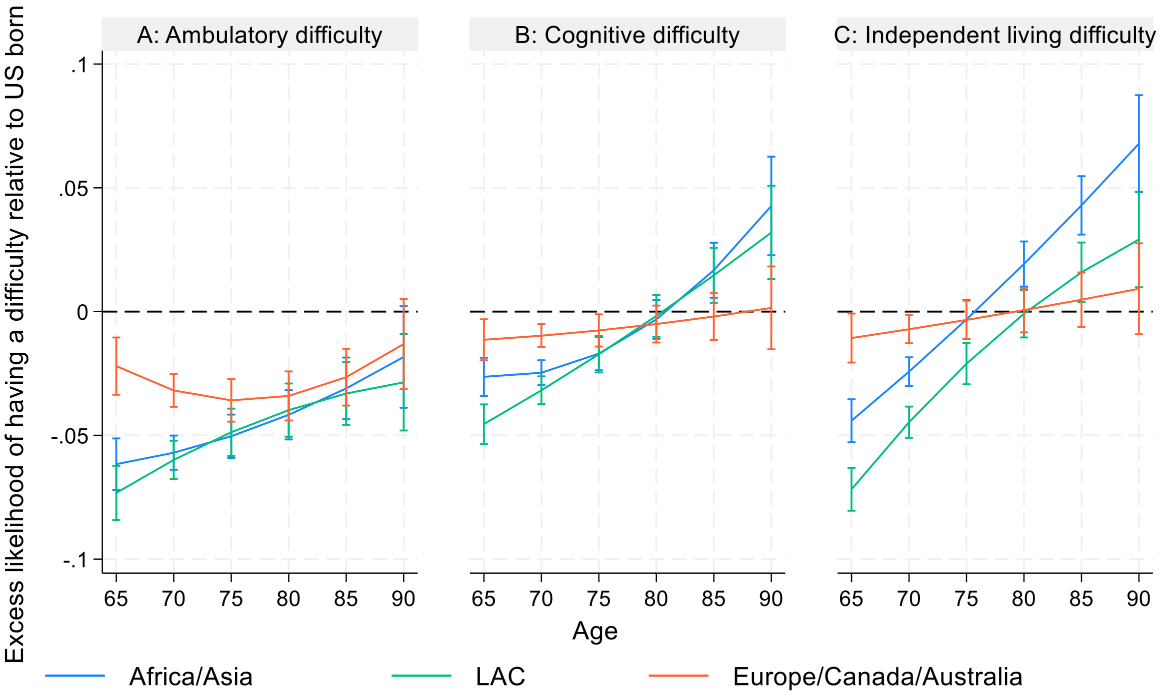

Source: Authors' calculation based on the American Community Survey using the age 65+ population in 2023.

Note: Estimates are based on predictive margins using linear regression of having a specific difficulty onto interactions of place of birth categories, age and age squared, control variables and county fixed effects. Three panels are based on three separate regressions.

Appendix Table 2: Regression results for Exhibit 3

	(1)	(2)	(3)
VARIABLES	cogdiff	ambuldiff	indepdiff
1.region_n	-0.0141**	-0.0306***	-0.00440
	[-2.196]	[-3.453]	[-0.566]
2.region_n	-0.0131***	-0.0524***	-0.00751**
	[-4.758]	[-14.41]	[-2.378]
3.region_n	-0.0208***	-0.0538***	-0.0313***
	[-7.345]	[-14.17]	[-9.765]
4.region_n	-0.00622***	-0.0269***	-0.000963
	[-2.583]	[-8.402]	[-0.332]
2.older	0.0451***	0.100***	0.0907***
	[44.91]	[71.26]	[75.54]
3.older	0.167***	0.312***	0.352***
	[75.29]	[119.3]	[139.1]
female	-0.00307***	0.0253***	0.0222***
	[-3.439]	[20.05]	[21.27]
married	-0.0294***	-0.0652***	-0.0447***
	[-32.76]	[-50.85]	[-42.22]
black	0.00686***	0.0307***	0.0112***
	[3.721]	[11.82]	[5.210]
other_race	0.00713***	0.0221***	0.00876***
	[3.482]	[8.011]	[3.767]
hispanic	-0.00406	-0.00521	-0.00912***
	[-1.503]	[-1.429]	[-2.988]
medicaid	0.119***	0.180***	0.164***
	[73.75]	[88.75]	[89.86]

medicare	0.00938***	0.0428***	0.0227***
	[6.483]	[19.98]	[13.70]
HS	-0.0502***	-0.0594***	-0.0638***
	[-23.54]	[-22.43]	[-26.77]
some_college	-0.0643***	-0.0766***	-0.0852***
	[-29.10]	[-27.26]	[-34.32]
CG	-0.0785***	-0.121***	-0.104***
	[-37.00]	[-45.36]	[-43.30]
veteran	0.00857***	0.0268***	-0.00285*
	[6.222]	[13.97]	[-1.833]
Constant	0.110***	0.192***	0.132***
	[42.47]	[56.27]	[45.68]
Observations	788,193	788,193	788,193
R-squared	0.079	0.126	0.155

Appendix Table 3: Regression results for Exhibit 4

```
mlogit living_arr_cat i.region_n##i.indepdiff  i.older female married black other_race hispanic
medicaid medicare  HS some_college CG veteran i.statefip [w=perwt], robust
(frequency weights assumed)

Iteration 0:  Log pseudolikelihood =  -35195317
Iteration 1:  Log pseudolikelihood =  -31691993
Iteration 2:  Log pseudolikelihood =  -30507752
Iteration 3:  Log pseudolikelihood =  -30413904
Iteration 4:  Log pseudolikelihood =  -30412180
Iteration 5:  Log pseudolikelihood =  -30412179
Iteration 6:  Log pseudolikelihood =  -30412179

Multinomial logistic regression                    Number of obs  = 59,325,594
                                                   Wald chi2(144) = 8429817.74
                                                   Prob > chi2    =     0.0000
Log pseudolikelihood = -30412179                   Pseudo R2      =     0.1359
```

living_arr_cat	Coefficient	Robust std. err.	z	P>|z|	[95% conf.	interval]
0	(base outcome)					
1						
region_n						
Africa	1.08812	.0043325	251.15	0.000	1.079629	1.096612
Asia	.9849798	.0019087	516.04	0.000	.9812388	.9887209
Latin America and the Caribbean	.7043743	.0017088	412.22	0.000	.7010252	.7077234
Europe/Canada/Australia	.1771852	.0021989	80.58	0.000	.1728753	.181495
1.indepdiff	.5709665	.0011257	507.19	0.000	.56876	.5731729
region_n#indepdiff						
Africa#1	-.0860538	.0117591	-7.32	0.000	-.1091013	-.0630063
Asia#1	-.1525388	.0033812	-45.11	0.000	-.1591659	-.1459117
Latin America and the Caribbean#1	-.2899652	.0030989	-93.57	0.000	-.296039	-.2838915
Europe/Canada/Australia#1	.002782	.0047716	0.58	0.560	-.0065702	.0121341
older						
2	-.0847222	.0008361	-101.33	0.000	-.086361	-.0830835
3	.1367122	.0013012	105.06	0.000	.1341619	.1392626
female	.1272829	.0007824	162.67	0.000	.1257493	.1288164
married	-.1165896	.0007416	-157.21	0.000	-.1180431	-.115136
black	.5086102	.0012063	421.62	0.000	.5062459	.5109745
other_race	.3303142	.0014725	224.32	0.000	.3274281	.3332003
hispanic	.3735282	.0017897	208.71	0.000	.3700204	.3770359
medicaid	.0050492	.0010231	4.94	0.000	.0030439	.0070545
medicare	-.4913623	.0014652	-335.35	0.000	-.4942341	-.4884906
HS	-.1641651	.0011859	-138.44	0.000	-.1664893	-.1618408
some_college	-.353715	.0013467	-262.65	0.000	-.3563545	-.3510754
CG	-.5224306	.0012908	-404.72	0.000	-.5249606	-.5199006
veteran	-.0493035	.001254	-39.32	0.000	-.0517613	-.0468458
statefip						
Alaska	.301838	.0086727	34.80	0.000	.2848399	.3188361
Arizona	-.0810113	.0037861	-21.40	0.000	-.0884319	-.0735907
Arkansas	-.1009494	.0049626	-20.34	0.000	-.110676	-.0912229
California	.2453304	.0031101	78.88	0.000	.2392347	.2514262
Colorado	-.0116554	.0042026	-2.77	0.006	-.0198923	-.0034184
Connecticut	.2451827	.0043101	56.89	0.000	.236735	.2536304
Delaware	.0061204	.0066693	0.92	0.359	-.0069513	.0191921
District of Columbia	-.5095579	.0107102	-47.58	0.000	-.5305495	-.4885664
Florida	-.0693943	.0031937	-21.73	0.000	-.0756538	-.0631348
Georgia	.1916666	.0035273	54.34	0.000	.1847533	.1985799
Hawaii	.5415291	.0052299	103.54	0.000	.5312787	.5517795
Idaho	.0346542	.0058196	5.95	0.000	.023248	.0460605
Illinois	.0047133	.0034548	1.36	0.172	-.0020579	.0114845

Indiana	-.1499818	.004051	-37.02	0.000	-.1579216	-.1420421
Iowa	-.568225	.0056383	-100.78	0.000	-.5792758	-.5571742
Kansas	-.3219031	.0053848	-59.78	0.000	-.3324571	-.3113491
Kentucky	-.0352869	.0043889	-8.04	0.000	-.043889	-.0266848
Louisiana	-.1320795	.0043712	-30.22	0.000	-.140647	-.1235121
Maine	-.2434258	.0065695	-37.05	0.000	-.2563018	-.2305498
Maryland	.2168881	.0038209	56.76	0.000	.2093993	.2243769
Massachusetts	.2192485	.0037709	58.14	0.000	.2118576	.2266394
Michigan	-.0548243	.0035955	-15.25	0.000	-.0618713	-.0477773
Minnesota	-.2363757	.0043204	-54.71	0.000	-.2448434	-.2279079
Mississippi	-.0762451	.0049421	-15.43	0.000	-.0859314	-.0665588
Missouri	-.1013339	.0040554	-24.99	0.000	-.1092822	-.0933855
Montana	-.2321311	.0074182	-31.29	0.000	-.2466705	-.2175918
Nebraska	-.517076	.0067955	-76.09	0.000	-.5303949	-.503757
Nevada	.2030971	.0044903	45.23	0.000	.1942962	.211898
New Hampshire	.1473024	.0060719	24.26	0.000	.1354017	.1592031
New Jersey	.23907	.0035479	67.38	0.000	.2321163	.2460237
New Mexico	-.1070783	.0051929	-20.62	0.000	-.1172562	-.0969005
New York	.1625759	.0032318	50.31	0.000	.1562417	.16891
North Carolina	-.0703746	.003584	-19.64	0.000	-.077399	-.0633501
North Dakota	-.6405828	.0108803	-58.88	0.000	-.6619077	-.6192578
Ohio	-.1091411	.0035505	-30.74	0.000	-.1161	-.1021823
Oklahoma	.0112091	.0045375	2.47	0.013	.0023157	.0201024
Oregon	.0475491	.0042938	11.07	0.000	.0391334	.0559648
Pennsylvania	.0731963	.0034089	21.47	0.000	.066515	.0798777
Rhode Island	.1987754	.0065672	30.27	0.000	.185904	.2116468
South Carolina	-.0536805	.0040531	-13.24	0.000	-.0616243	-.0457366
South Dakota	-.2174627	.0084303	-25.80	0.000	-.2339858	-.2009395
Tennessee	.0533463	.003846	13.87	0.000	.0458083	.0608844
Texas	.128413	.0031938	40.21	0.000	.1221532	.1346728
Utah	.3883911	.0050157	77.44	0.000	.3785606	.3982217
Vermont	-.3834673	.0097856	-39.19	0.000	-.4026467	-.364288
Virginia	.1116462	.0036522	30.57	0.000	.104488	.1188045
Washington	.0697362	.0037996	18.35	0.000	.0622891	.0771833
West Virginia	-.0085516	.0055551	-1.54	0.124	-.0194394	.0023363
Wisconsin	-.2856863	.0042534	-67.17	0.000	-.2940228	-.2773497
Wyoming	-.4007387	.0106881	-37.49	0.000	-.421687	-.3797905
_cons	-1.190986	.003468	-343.42	0.000	-1.197783	-1.184189
2						
region_n						
Africa	-.4375535	.0305816	-14.31	0.000	-.4974924	-.3776147
Asia	-.5907352	.0115824	-51.00	0.000	-.6134364	-.568034
Latin America and the Caribbean	-.0255858	.0088348	-2.90	0.004	-.0429016	-.00827
Europe/Canada/Australia	-.3153549	.0099447	-31.71	0.000	-.3348461	-.2958637
1.indepdiff	2.930547	.0022795	1285.59	0.000	2.926079	2.935014
region_n#indepdiff						
Africa#1	-.5241536	.037111	-14.12	0.000	-.5968898	-.4514173
Asia#1	-.9387257	.0132938	-70.61	0.000	-.9647811	-.9126703
Latin America and the Caribbean#1	-.668744	.0098535	-67.87	0.000	-.6880565	-.6494315
Europe/Canada/Australia#1	-.8186938	.0119938	-68.26	0.000	-.8422013	-.7951864
older						
2	.412168	.002105	195.80	0.000	.4080422	.4162938
3	.8130593	.0024469	332.28	0.000	.8082634	.8178552
female	-.5466189	.0020587	-265.51	0.000	-.5506539	-.5425838
married	-1.184311	.002311	-512.47	0.000	-1.18884	-1.179782
black	-.1483999	.002897	-51.22	0.000	-.154078	-.1427218
other_race	-.2995648	.0039568	-75.71	0.000	-.3073199	-.2918097
hispanic	-.4465474	.0049953	-89.39	0.000	-.456338	-.4367569
medicaid	1.707194	.0019658	868.43	0.000	1.703341	1.711047
medicare	-1.544712	.0045476	-339.68	0.000	-1.553625	-1.535799
HS	.0281135	.0027651	10.17	0.000	.022694	.033533
some_college	-.0325873	.0032942	-9.89	0.000	-.0390437	-.0261308
CG	-.0601585	.0032371	-18.58	0.000	-.0665031	-.0538139

veteran	-.4082007	.0031612	-129.13	0.000	-.4143966	-.4020047
statefip						
Alaska	.553122	.0227226	24.34	0.000	.5085866	.5976574
Arizona	-.0396397	.009669	-4.10	0.000	-.0585907	-.0206888
Arkansas	.1063871	.0108181	9.83	0.000	.0851839	.1275902
California	.3976191	.0072926	54.52	0.000	.3833259	.4119124
Colorado	.2234964	.0101851	21.94	0.000	.2035339	.2434589
Connecticut	.1388554	.0098595	14.08	0.000	.1195311	.1581797
Delaware	-.2333313	.0187003	-12.48	0.000	-.2699833	-.1966793
District of Columbia	.1287117	.0192978	6.67	0.000	.0908888	.1665347
Florida	.186355	.0074671	24.96	0.000	.1717198	.2009902
Georgia	-.1656249	.0089468	-18.51	0.000	-.1831603	-.1480895
Hawaii	.161776	.0146652	11.03	0.000	.1330326	.1905193
Idaho	.3600844	.0132168	27.24	0.000	.3341799	.3859889
Illinois	.3396353	.007976	42.58	0.000	.3240026	.3552679
Indiana	.2776053	.0085609	32.43	0.000	.2608262	.2943843
Iowa	.7335259	.0100226	73.19	0.000	.7138819	.7531698
Kansas	.6249403	.0105334	59.33	0.000	.6042952	.6455854
Kentucky	.0059831	.0096995	0.62	0.537	-.0130277	.0249938
Louisiana	-.1197521	.0099797	-12.00	0.000	-.1393119	-.1001922
Maine	.5432531	.0133892	40.57	0.000	.5170107	.5694955
Maryland	.1991996	.0095094	20.95	0.000	.1805616	.2178377
Massachusetts	.2477441	.0086128	28.76	0.000	.2308633	.2646248
Michigan	.0768661	.0083323	9.23	0.000	.0605351	.0931971
Minnesota	.639899	.0088679	72.16	0.000	.6225182	.6572797
Mississippi	-.0793628	.0108477	-7.32	0.000	-.1006238	-.0581018
Missouri	.3277243	.0088846	36.89	0.000	.3103108	.3451378
Montana	.4483962	.0154544	29.01	0.000	.418106	.4786863
Nebraska	.404126	.0128849	31.36	0.000	.3788721	.4293799
Nevada	-.3277321	.0141265	-23.20	0.000	-.3554196	-.3000446
New Hampshire	.7359839	.0131998	55.76	0.000	.7101129	.761855
New Jersey	.2609341	.0086799	30.06	0.000	.2439217	.2779464
New Mexico	-.3998052	.0147631	-27.08	0.000	-.4287403	-.3708701
New York	.2260395	.0074378	30.39	0.000	.2114617	.2406174
North Carolina	-.1070629	.0086014	-12.45	0.000	-.1239214	-.0902045
North Dakota	.7907971	.0172621	45.81	0.000	.7569641	.8246302
Ohio	.3529067	.0078044	45.22	0.000	.3376104	.368203
Oklahoma	.2053618	.0105245	19.51	0.000	.1847341	.2259894
Oregon	.1512279	.0100723	15.01	0.000	.1314866	.1709692
Pennsylvania	.2748448	.0077421	35.50	0.000	.2596705	.2900191
Rhode Island	.3942422	.0141188	27.92	0.000	.3665699	.4219144
South Carolina	-.1293044	.0099668	-12.97	0.000	-.1488391	-.1097698
South Dakota	.7015843	.0161386	43.47	0.000	.6699533	.7332153
Tennessee	-.1211963	.0092561	-13.09	0.000	-.139338	-.1030546
Texas	.0794202	.0075901	10.46	0.000	.0645438	.0942966
Utah	-.0419611	.0151266	-2.77	0.006	-.0716087	-.0123135
Vermont	.2393816	.0191157	12.52	0.000	.2019154	.2768477
Virginia	.2323172	.0088919	26.13	0.000	.2148893	.2497451
Washington	.1714497	.009215	18.61	0.000	.1533887	.1895107
West Virginia	-.2594134	.0128875	-20.13	0.000	-.2846724	-.2341543
Wisconsin	.3505708	.0091029	38.51	0.000	.3327294	.3684122
Wyoming	-.065976	.0218869	-3.01	0.003	-.1088736	-.0230784
_cons	-3.170991	.0086863	-365.06	0.000	-3.188016	-3.153966

```
. margins , dydx(indepdiff) over(region_n)

Average marginal effects                          Number of obs = 59,325,594
Model VCE: Robust

dy/dx wrt: 1.indepdiff
Over:      region_n

1._predict: Pr(living_arr_cat==0), predict(pr outcome(0))
2._predict: Pr(living_arr_cat==1), predict(pr outcome(1))
3._predict: Pr(living_arr_cat==2), predict(pr outcome(2))

------------------------------------------------------------------------------------------
                           |            Delta-method
```

	dy/dx	std. err.	z	P>|z|	[95% conf.	interval]
0.indepdiff	(base outcome)					
1.indepdiff						
_predict#region_n						
1 0	-.158618	.0001872	-847.12	0.000	-.158985	-.1582511
1#Africa	-.1310947	.0027057	-48.45	0.000	-.1363977	-.1257917
1#Asia	-.1086476	.0007661	-141.82	0.000	-.1101492	-.1071461
1#Latin America and the Caribbean	-.0868201	.0006887	-126.06	0.000	-.08817	-.0854702
1#Europe/Canada/Australia	-.1219533	.0008138	-149.86	0.000	-.1235483	-.1203584
2 0	.0568218	.0001647	345.08	0.000	.0564991	.0571446
2#Africa	.0945242	.0027192	34.76	0.000	.0891946	.0998537
2#Asia	.0888813	.0007647	116.23	0.000	.0873826	.09038
2#Latin America and the Caribbean	.0477062	.0006863	69.51	0.000	.0463611	.0490513
2#Europe/Canada/Australia	.0789564	.0007847	100.62	0.000	.0774184	.0804943
3 0	.1017962	.0001149	885.82	0.000	.101571	.1020214
3#Africa	.0365705	.0007854	46.56	0.000	.0350312	.0381099
3#Asia	.0197663	.0001626	121.59	0.000	.0194477	.0200849
3#Latin America and the Caribbean	.0391139	.0002136	183.12	0.000	.0386952	.0395325
3#Europe/Canada/Australia	.042997	.0003038	141.54	0.000	.0424016	.0435924

Note: dy/dx for factor levels is the discrete change from the base level.
